# Finite Element Analysis of Axial Compression Steel Members Strengthened with Unbonded CFRP Laminates

**DOI:** 10.3390/ma13163540

**Published:** 2020-08-11

**Authors:** Fengky Satria Yoresta, Phan Viet Nhut, Yukihiro Matsumoto

**Affiliations:** 1 Department of Architecture and Civil Engineering, Toyohashi University of Technology, Toyohashi, Aichi 441-8580, Japan; phan.viet.nhut.yu@tut.jp (P.V.N.); y-matsum@ace.tut.ac.jp (Y.M.); 2 Engineering and Building Design Laboratory, Bogor Agricultural University, Bogor 16680, Indonesia; 3 Department of Civil Engineering, The University of Danang - University of Technology and Education, Danang City 550000, Viet Nam

**Keywords:** unbonded CFRP, finite element, axial compression, steel members

## Abstract

This paper presented a non-linear finite element (FE) analysis to investigate the potential of unbonded carbon fiber-reinforced polymers (CFRP) strengthening in improving the axial compression performance of steel members. The FE model was firstly developed and validated against experimental works. Four parameters considered in the parametric study were the number of CFRP layers, CFRP length, slenderness ratio, and elastic modulus of CFRP. It was confirmed that the unbonded CFRP strengthening method is effective at enhancing the load-carrying capacity as well as delaying the overall buckling of the axial steel members. The strength increase is highly affected by the first three parameters. In addition, the method of an equivalent slenderness ratio can be used for strength design.

## 1. Introduction

Strengthening steel structures with adhesively bonded (or externally bonded) carbon fiber-reinforced polymers (CFRP) has been considerably developed in recent years. Many researchers, through their experimental, analytical, and/or numerical investigations, showed that this method of strengthening is able to improve the performance of steel structures. As confirmed by Kabir et al. [1], the bending capacity of CHS beams can increase up to 37% after the beams are bonded with CFRP. The effectiveness of the strengthening for the ultimate flexure capacity of steel I-beams was found to be about 10% by Siwowski and Siwowska [2]. Linghoff et al. [3] also reported the increase of bending strength of steel I-beams by up to about 20%. In improving the fatigue life of steel structures, Liu et al. [4] developed a simple analytical model for estimating the fatigue life of CFRP repaired steel plates. The fatigue life of cracked steel plates can be greatly increased using adhesively bonded CFRP with the improving factors ranging from 3.26 to 7.47 [5]. Similar findings are also reported by Wang et al. [6] with the extension ratio of fatigue life 3.5 to 11.3. Sayed-Ahmed et al. [7], Ritchie et al. [8], Shaat and Fam [9], and Imran et al. [10] concluded that adhesively bonded CFRP is effective for strengthening steel members experiencing compressive load. The externally bonded CFRP method can also improve the performance of steel members subjected to torsional loading [11,12,13] and steel shear wall [14,15].

Even though the performance of steel structures can be much improved, strengthening steel using adhesively bonded CFRP is susceptible to its performance degradation because the bonding strength between CFRP and steel, where this method is highly dependent, can be influenced by many factors. This becomes the weakest point of this strengthening method. Long-term environmental exposures lead to the bond strength decrease due to effects of temperature [16,17,18], moisture [19], and ultraviolet (UV) exposure [20]. The bond strength will also be affected by the quality of steel surface treatment produced by the workers who work on it, as their skills are different. Steel surface treatment is a necessity to obtain a good bond between CFRP and steel, but it is not a simple task. It becomes difficult, especially when the strengthening will be applied to existing structures. Steel surface treatment will take longer and require more effort, which will lead to higher construction costs.

As an alternative to the adhesively bonded CFRP, which have limitations as such those mentioned above, an unbonded CFRP strengthening method is proposed [21]. This strengthening method does not rely on CFRP–steel bonding strength, but the flexural rigidity of CFRP becomes the most important point. The unbonded condition is obtained by placing an unbonded material between the steel and CFRP so that troublesome steel surface treatments are no longer required. It may lead to the installation process becoming much faster. This will also allow CFRP to be used for a long time without having to worry about the bond strength degradation of steel and CFRP. Thus, it will reduce construction costs. The finite element (FE) study presented in this paper is part of a research program conducted by the authors investigating the applicability of unbonded CFRP for strengthening steel. Experimental tests have been carried out at the Structural Engineering laboratory, Toyohashi University of Technology, and these are presented in Yoresta et al. [21]. In this study, steel bars are modeled in finite element and partially strengthened using unbonded CFRP to further investigate the potential of this strengthening method in improving the buckling performance of axial compression steel members. The developed finite element model is firstly followed by validation against the experimental test results. Afterwards, a parametric study is conducted to examine the effect of several parameters on the buckling performance of the members. Finally, the applicability of the equivalent slenderness ratio method for determining the allowable compressive stress of the members is presented.

## 2. Summary of Experimental Investigation

This section presents a brief summary of the experimental study, whereas more details can be found in Yoresta et al. [21]. The experimental study consisted of testing a total of eight pinned-end slender steel bar specimens, having yield strength (*F*_y_) of 328 MPa, under compressive loading. All specimens consist of steel, with a diameter (*D*) of 32 mm, which is divided into two types of length, namely 560 mm and 760 mm. These two lengths correspond to slenderness ratios of 70 and 95 respectively, which are referred to as specimens Group 1 and 2 hereafter. Each of them has two CFRP lengths and a bare steel acting as a control unstrengthened specimen. The CFRP are positioned at the mid-span of steel and fully covering its cross-section. It is determined that CFRP with the length of 260 mm and 340 mm belong to the specimen with a slenderness ratio of 70, while the length of 360 mm and 460 mm belong to the specimen with a slenderness ratio of 95. Two specimens with a CFRP length of 260 mm are distinguished by 26 and 28 plies of carbon fiber respectively, while the other two specimens with 340 mm CFRP length are distinguished by 27 and 28 plies of carbon fiber. Meanwhile, steel with CFRP lengths of 360 mm and 460 mm respectively consist of one specimen with the same 30 plies of carbon fiber. All specimens are labeled with identification codes. For example, BL56F26-1 indicates specimen number 1 with 560 mm of buckling length (BL56) and 260 mm length of carbon fiber (F26).

The CFRP laminates are made of bidirectional high-strength carbon fiber sheets, BT70-20 ([0/90] fiber orientation angle), having a thickness, tensile strength, and elastic modulus of 0.112 mm, 2.9 GPa, and 230 GPa, respectively. In the strengthening procedure, a layer of peel ply was first installed directly onto the steel surface before applying the carbon fiber sheet to prevent direct contact between the steel and CFRP as well as providing an unbonded condition between them. No steel surface treatments (e.g., sand blasting, hand grinding, or grit blasting) are applied, because it is completely not necessary for this strengthening method. Epoxy resin E205 (produced by Konishi Co.,Ltd., Osaka, Japan) is used, but it is only intended to bond the carbon fibers together, not for bonding carbon fibers onto the steel surface, as it has been separated by a peel ply. The resin is a material with ultra-low viscosity, high strength, and excellent durability. In the process of molding CFRP, the vacuum-assisted resin transfer molding (VaRTM) technique is adopted as an attempt to obtain high-quality and stable material properties of the CFRP as well as zero clearance between the CFRP and steel.

All specimens are tested up to buckling using a compression testing machine having a capacity of 2000 kN. The experimental results showed that unbonded CFRP strengthening successfully delays the overall buckling of the steel members without failure in CFRP. The compressive strength is also increased together with changes in the curvature position from the mid-height of members to the edges of the CFRP. The load versus lateral displacements behavior, ultimate loads, and failure modes of the members obtained from the experimental test can be seen and will be presented in sections throughout this paper.

## 3. Numerical Work 

### 3.1. Finite Element Model

A three-dimensional (3D) finite element model is developed to predict the behavior of the pinned-ends axial compression steel members partially strengthened with unbonded CFRP laminates. The results obtained from finite element analysis are compared with previous experimental work [21] for verification purposes. After the correctness of the proposed model is verified, a numerical parametric study is conducted for varied unbonded CFRP strengthening schemes. A commercial finite element (FE) package, LUSAS Version 14.7 [22], is used for implementations. The matrix of experimental specimens to be verified and designation for the finite element models used in the current study are shown in Table 1. The suffix “FE” is utilized in labeling the finite element models to distinguish them from the experimental specimens.

**Figure 1:**
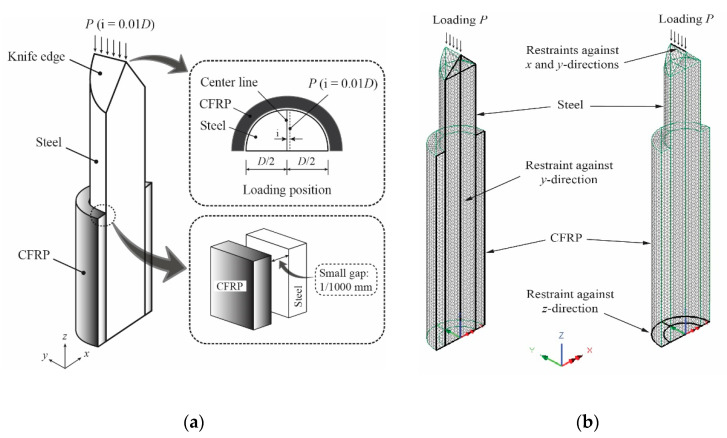
Features of the finite element (FE) model along with the boundary conditions and loading of steel members strengthened with unbonded carbon fiber-reinforced polymers (CFRP): (**a**) Summary of analytical condition; (**b**) Meshing condition.

Two symmetrical planes have been identified for along and across the geometric model. This condition allows the finite element model in the current study to be developed in one quarter where the specimen is reduced to only half the width and half the length, respectively. This is an effort that is intended to get the advantage of symmetric properties of specimens in terms of geometry, material properties, loading, and also boundary conditions. Moreover, building a quarter model of the structure can also reduce the total number of elements and thus shorten the computational time significantly. Figure 1 shows the geometrical features of the quarter FE model developed.

### 3.2. Meshing and Elements

**Figure 2:**
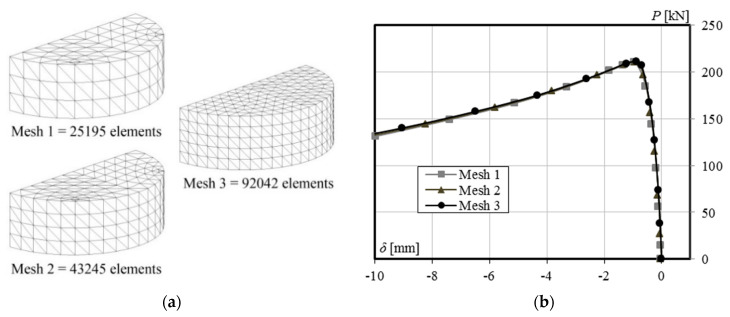
Meshing system: (**a**) Mesh density; (**b**) Mesh validation.

Modeling the finite element model involves creating two parts: steel and CFRP. All the parts are modeled by using four-noded solid continuum elements (TH4). The thickness and length of the CFRP are respectively assigned to be same with the measured values obtained from the experiment, as given in Table 1. An irregular mesh type with a relatively fine mesh density is applied for both CFRP and steel. However, in order to get good results but also not to spend too much time on analysis, numerical simulations are firstly conducted to the bare steel (control unstrengthened specimen, NS56BL) by applying three different element sizes, as shown in Figure 2a. Mesh 1 has 25,195 elements, mesh 2 has 43,245 elements, and mesh 3 has 92,042 elements. Then, an identical result on the load-lateral displacement behavior can be confirmed from Figure 2b. As such, mesh 1 with 25,195 elements is used for all analyses in this study, as its computer run-time will be much smaller than that of the other larger mesh elements.

### 3.3. Material Properties

**Figure 3:**
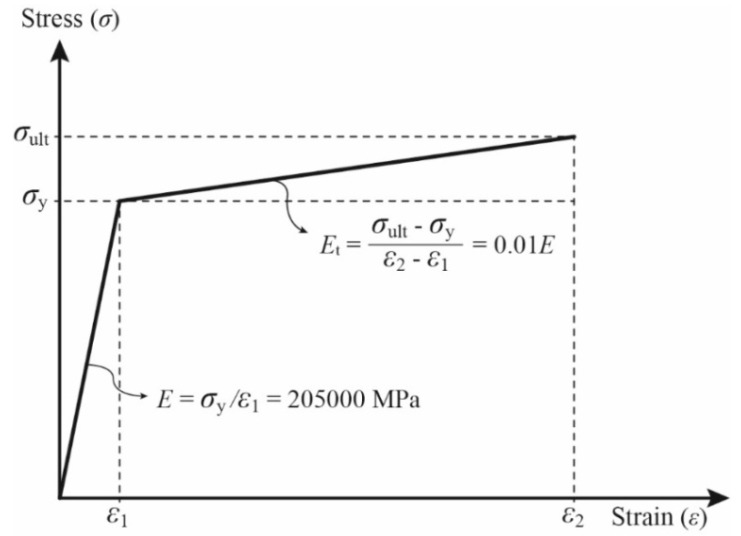
Bilinear steel material model.

A bilinear stress–strain behavior is used to take into account the non-linearity (plasticity) of steel (Figure 3). Here, the isotropic hardening rule and Von Mises yield criterion are adopted. The yield strength, modulus of elasticity, and ultimate tensile strength are the same as those used in the experiment (328 MPa, 205 Gpa, and 459 Mpa, respectively). The tangent modulus after the initial yielding of steel (*E*_t_) is assumed to be 1% of its elastic modulus (0.01*E*), and the Poisson’s ratio is taken to be equal to 0.3.

**Figure 4:**
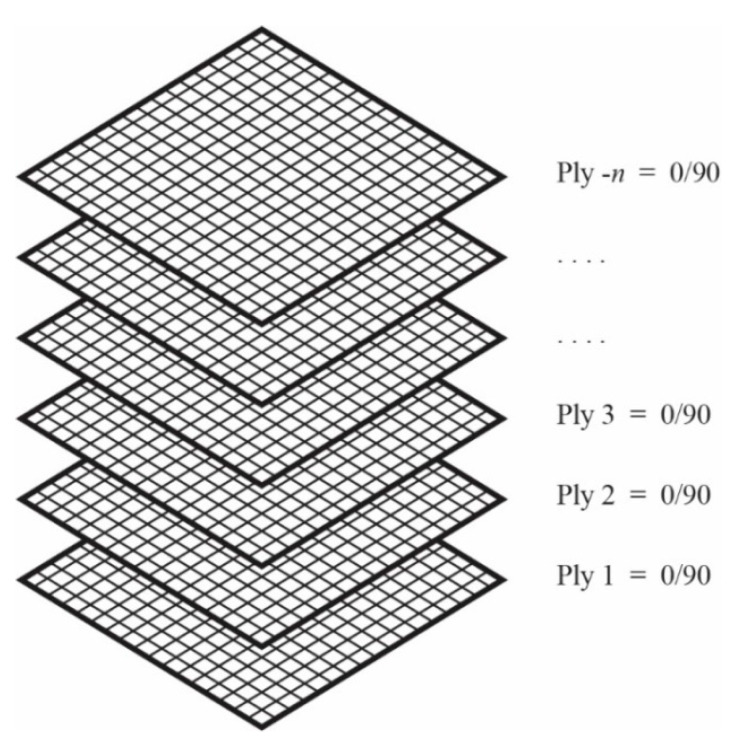
Stacking sequence of carbon fiber.

In this study, CFRP is considered to act as a stiffener that solely sustains bending about the longitudinal axes due to the buckling of the structure. Besides that, it is made from carbon fiber layers having balanced fiber orientations in the lamination stack-up sequence (see Figure 4) so that it falls within the category of quasi-isotropic laminates. The many layers of carbon fiber utilized also make it become thicker in size. These conditions make the elastic properties not be defined for each direction separately. The CFRP is assumed to behave as an elastic isotropic material. This is also the reason behind choosing the solid element for its modeling, as previously mentioned. The elastic modulus of CFRP is strongly dependent on the fiber volume contents (*V*_f_) and determined by applying the Classical Laminated Theory [23] once the actual thickness of the CFRP in the strengthened members had been obtained after the demolding process in the experiment. The elastic modulus value (*E*_CFRP_) and Poisson’s ratios (*ν*) of the CFRP used in the current finite element analysis are given in Table 1, which are based on the assumption that the elastic modulus and Poisson’s ratio of epoxy resin used are 4 Gpa and 0.3, respectively.

### 3.4. Support Conditions

As previously mentioned, the FE model is developed in one-quarter of the specimen because of the double symmetry of the members. To reach this goal in length, the bottom end of the member is kept restrained for translation in the *z*-direction only. Meanwhile, the other end is kept restrained for the *x* and *y*-direction translation to allow loading the application along the length of the member (*z*-direction). Then, to achieve symmetric width, all the nodes along the longitudinal symmetry plane are kept restrained in the *y*-direction. This led to allowing out-of-plane buckling deformation occurring in the *x*-direction only, as desired (See Figure 1).

### 3.5. Unbonded Condition

A very small gap of 1/1000 mm with no-friction slidelines application on both the CFRP and steel surface is defined to establish an unbonded condition between these two materials (Figure 1). This assumption was applied because very small strain values are experimentally observed at the CFRP before buckling occurred (i.e., below the ultimate loads), and it is relatively constant until reaching the ultimate load. 

### 3.6. Load Application 

The load is assigned at position 1% of the steel diameter (0.01*D*) in the positive direction of the x-axis. This assumed value is determined after several numerical trials for representing unmeasured imperfections in all the real specimens in experimental works, which is usually due to the different values in the initial straightness, inevitable misalignment within the test setup, or a combination of these factors. The load *P* is applied as a distributed pressure over the length of the knife edge (Figure 1), which corresponds to the load application in laboratory tests. In analysis, the arc-length control method [24] is used to control the load application. This is a better choice for buckling problems, as it prevents instability (divergence) in non-linear iterative processes, even if the slope is zero (or negative) [25]. A convergence can be achieved for near-limit points because the load level is not constant during increments. 

### 3.7. Model Validation

**Figure 5:**
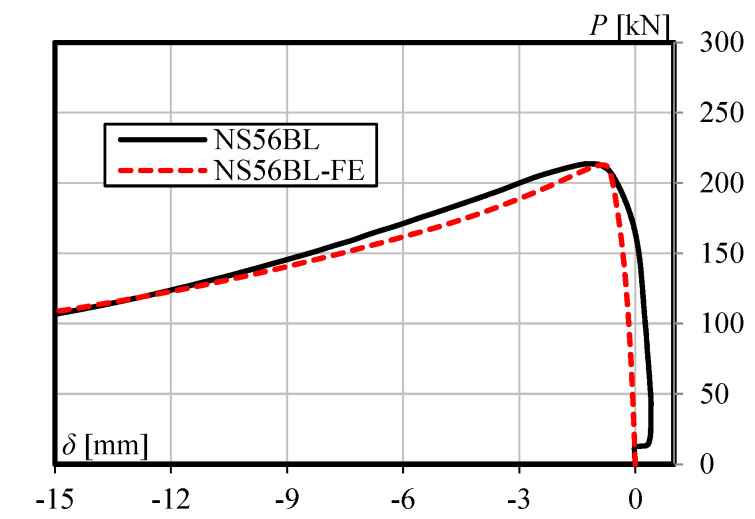
Load versus lateral mid-height deflection response for specimen NS56BL.

**Figure 6:**
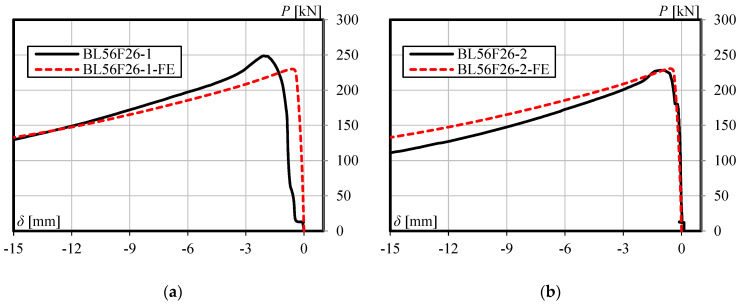
Load versus lateral mid-height deflection response for specimen having a strengthening length of 260 mm: (**a**) BL56F26-1, and (**b**) BL56F26-2.

**Figure 7:**
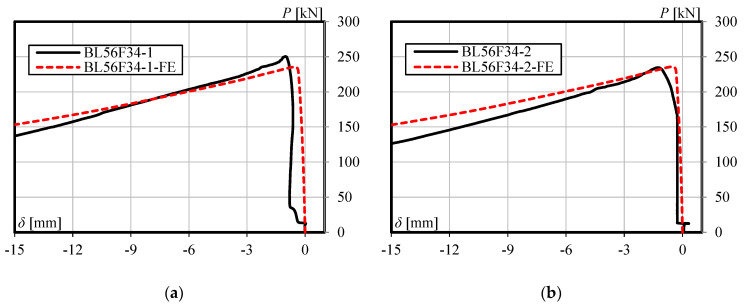
Load versus lateral mid-height deflection response for specimen having a strengthening length of 340 mm: (**a**) BL56F34-1, and (**b**) BL56F34-2.

**Figure 8:**
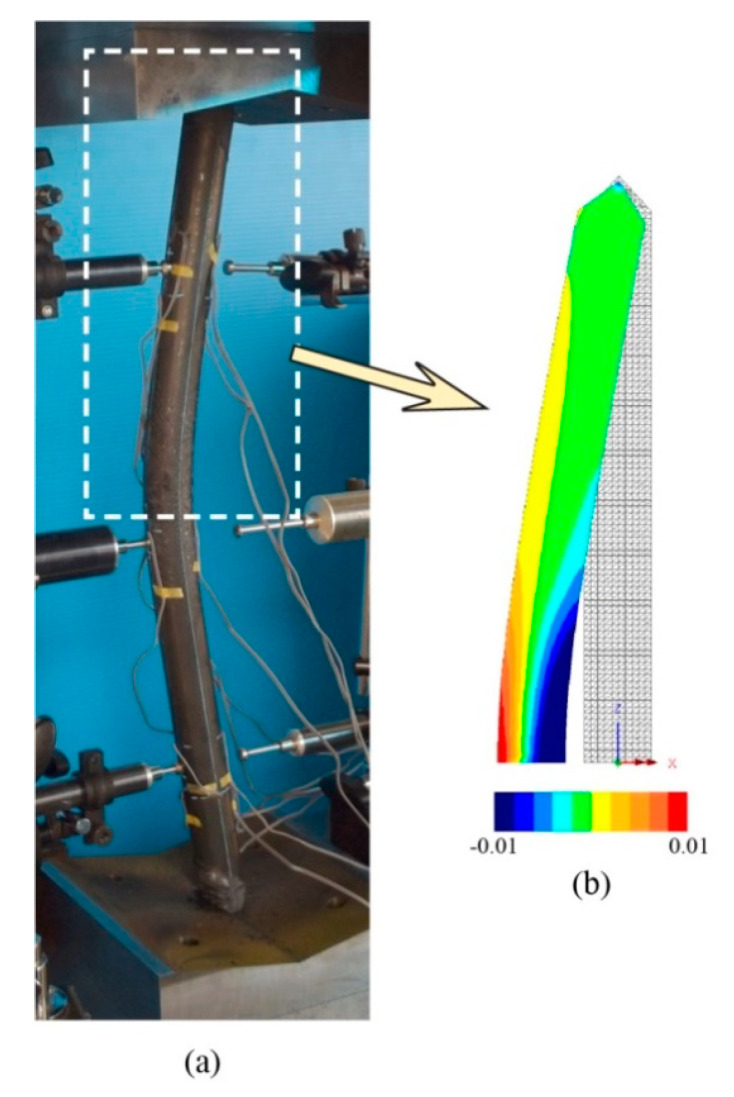
Failure mode of control unstrengthened specimen: (**a**) NS56BL and (**b**) NS56BL-FE along with longitudinal (*z*) strain contour.

**Figure 9:**
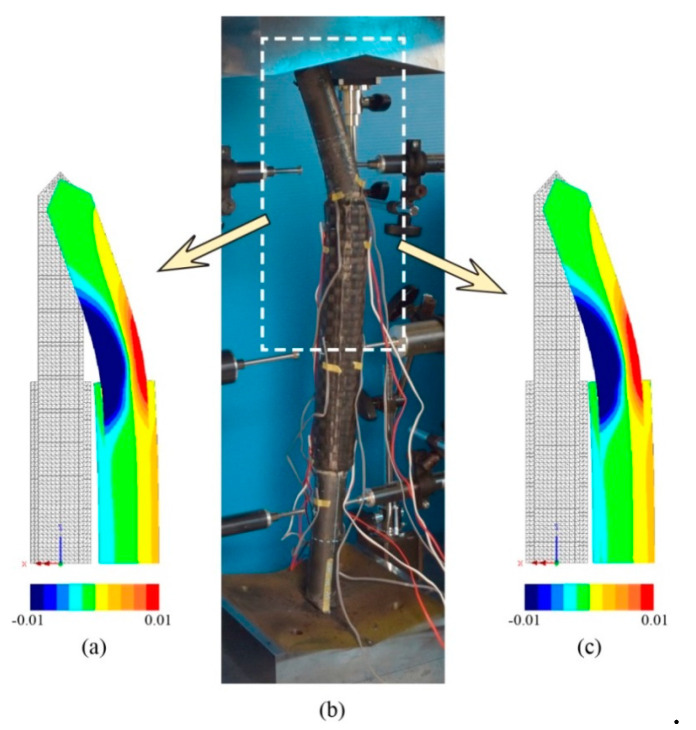
Failure mode of specimen with 260 mm strengthening length: (**a**) BL56F26-1-FE along with longitudinal (*z*) strain contour, (**b**) typical experimental failure for BL56F26-1 and BL56F26-2, and (**c**) BL56F26-2-FE along with longitudinal (*z*) strain contour.

**Figure 10:**
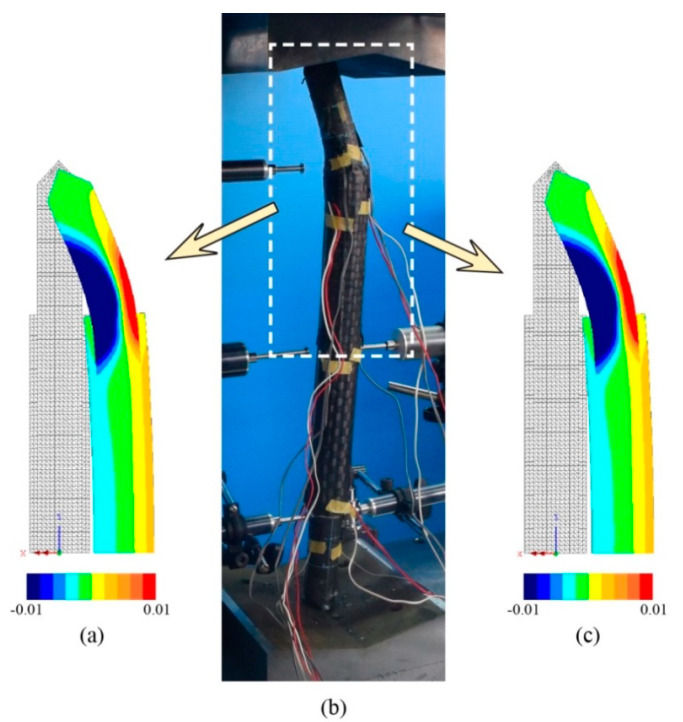
Failure mode of specimen with 340 mm strengthening length: (**a**) BL56F34-1-FE along with longitudinal (*z*) strain contour, (**b**) typical experimental failure for BL56F34-1 and BL56F34-2, and (**c**) BL56F34-2-FE along with longitudinal (*z*) strain contour.

The accuracy of the finite element model is verified by comparing with the experimental results. Table 2 shows the comparison of load-carrying capacity between FE analysis and the experimental test. Furthermore, Figure 5, Figure 6 and Figure 7 shows the load versus lateral mid-height deflection response of specimens from the experimental measurements and finite element prediction. It is clear from Table 2 and Figure 5, Figure 6 and Figure 7 that a good agreement between the experimental and numerical results can be observed. The difference of load-carrying capacity is confirmed to be less than 10%. The variation of differences is most likely due to the application of the same imperfection for all specimens. For further validation, the failure modes of the FE models are also compared with those obtained from the experiment. The buckled shapes of each model predicted by finite element analysis including strain contours in the longitudinal (*z*) direction (at 20 mm lateral displacement of mid-height) and the typical failure mode of steel members having strengthening lengths of 260 mm (BL56F26) and 340 mm (BL56F34) are shown in Figure 8, Figure 9 and Figure 10. It is clear from the figures that the control specimen undergoes a change in curvature at the middle height, and all the strengthened specimen experiences it around the edge of the CFRP, which is similar to the buckling failure modes observed in the tests. Thus, it is concluded that the proposed FE models are valid and can be reliably used as a numerical tool to predict the axial compression response of steel members strengthened with unbonded CFRP laminates. 

## 4. Parametric Study

After validating the numerical modeling procedure, a parametric study is carried out by creating 33 models with the same material properties and cross-sectional dimension of steel as those used in the experimental works. The models are developed based on the stiffening design requirement [21] to investigate the effect of some influential parameters, i.e., (1) the number of CFRP layers, (2) the length of the CFRP, (3) the slenderness ratio, and (4) the elastic modulus of the CFRP, on the buckling behavior and ultimate strength of axial compression of a steel member strengthened with fully cross-sectional-jacketed unbonded CFRP.

Excluding the control specimen, the following labeling system was built to facilitate identification of the models. The first number indicates the slenderness ratio of the initial steel member (bare steel, namely 120, 100, and 70), while the second number represents the length of CFRP strengthening (385, 480, and 625). The combination ‘number–letter’ following these two numbers is used for identifying the number of CFRP layers involved. Then, the last term (E1 and E2) indicates the values of elastic modulus of the CFRP, which correspond to the assumed fiber volume content (50% and 55%) used herein, i.e., 62.63 GPa and 68.72 GPa, respectively. For instance, 120-385-20L-E2 identifies a specimen model that has a slenderness ratio of 120, strengthened with 20 layers of CFRP of 385 mm length, which have an elastic modulus of 68.72 GPa. For control specimens, labeling only uses the word “control” followed by the slenderness ratio—for example, bare steel with a slenderness ratio of 100: “Control-100”. It should be noted that the use of 50% and 55% fiber content are based on the experimental findings for the estimation of the lowest and highest value of fiber content in CFRP resulting from the VaRTM molding process.

**Figure 11:**
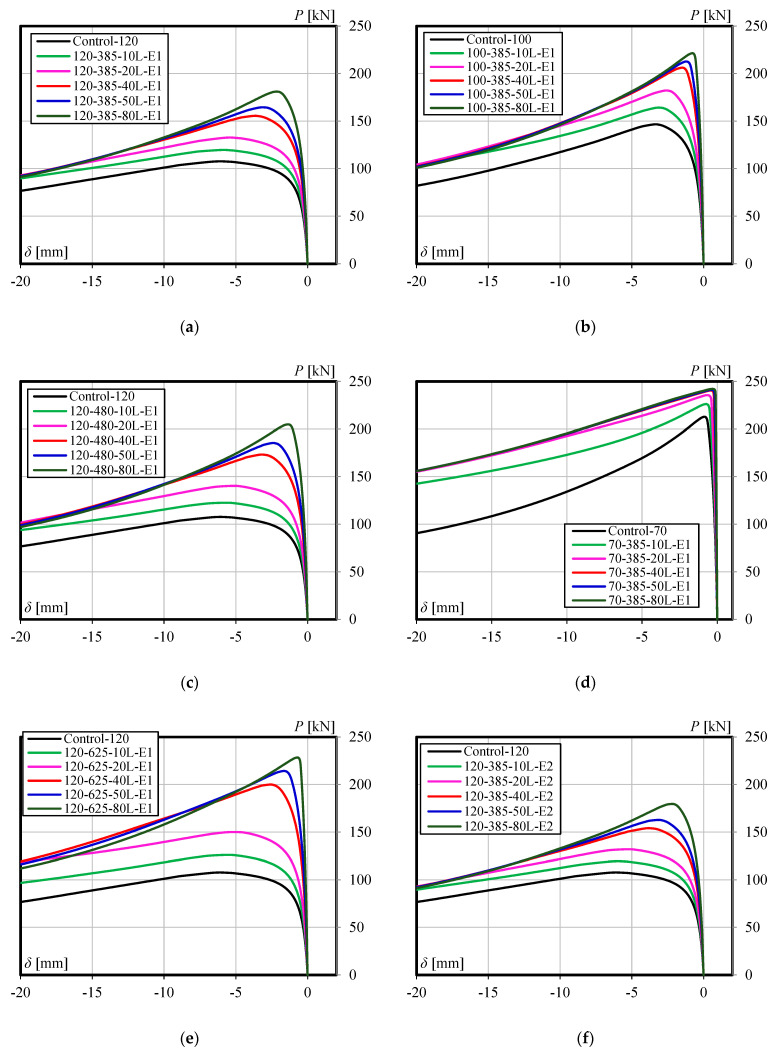
Load-lateral displacement behavior of all specimens: (**a**), (**c**), and (**e**) The member having slenderness ratio 120 with CFRP length of 385mm, 480mm, and 625mm, respectively; (**b**) and (**d**) The member having CFRP length of 385mm with slenderness ratio 100 and 70, respectively; (**f**) The member having slenderness ratio 120, CFRP length of 385mm, and CFRP modulus 68.72 GPa.

**Figure 12:**
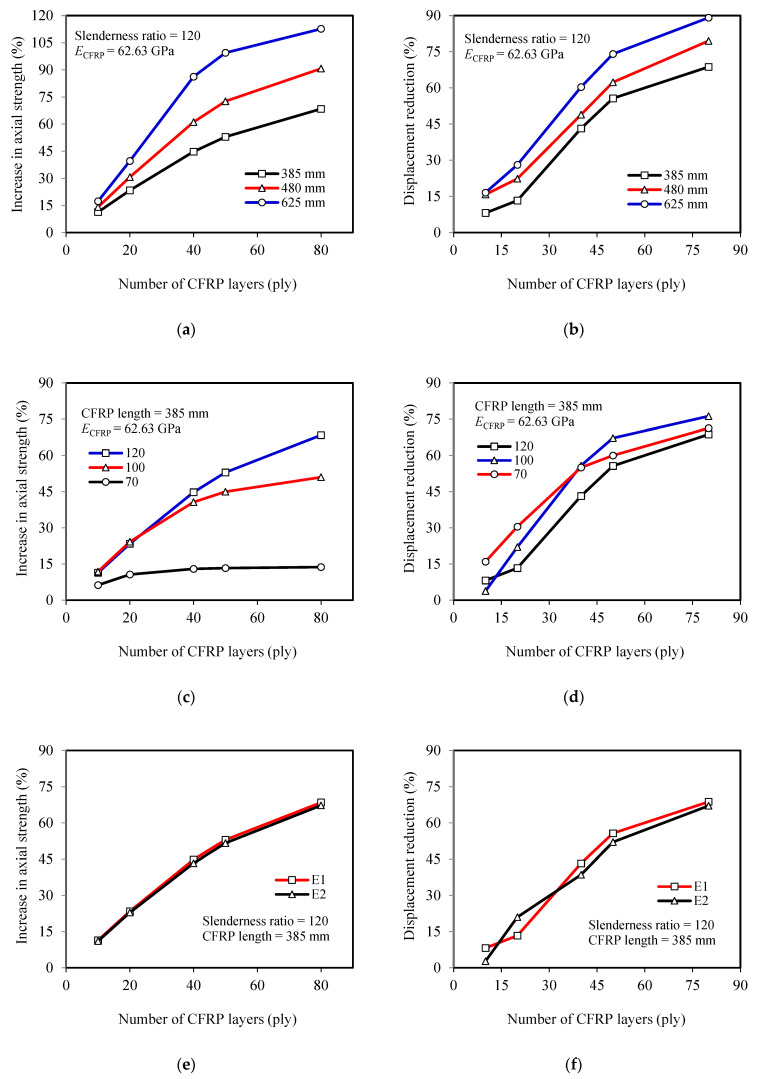
Parametric study results: (**a**), (**c**), and (**e**) Effect of CFRP length, steel’s slenderness ratio, and CFRP’s elastic modulus respectively on strength increase; (**b**), (**d**), and (**f**) Effect of CFRP length, steel’s slenderness ratio, and CFRP’s elastic modulus respectively on displacement reduction.

The results obtained from the finite element simulation, including the maximum loads and lateral displacement at mid-height at maximum loads, are summarized in Table 3. The table also presents the percentage increases in maximum loads as well as decreases in lateral displacements at maximum loads over the unstrengthened model. The load versus lateral displacement responses of all specimens are shown in Figure 11. Figure 12 presents a summary of the parametric study, which shows the effect of each parameter investigated on the percentage increases of compression strength and decreases in lateral displacements at maximum loads.

### 4.1. Effect of Number of CFRP Layers

The effect of the number of CFRP layers on the increase of compressive strength and lateral displacement reduction of members can be seen in Figure 12a,c,e and Figure 12b,d,f, respectively. Moreover, it is also demonstrated by all the graphs in Figure 11. It is clear from Figure 11 and Figure 12a,c,e that the compressive strength increases along with the increasing number of CFRP layers, but the rate is not linear. The increase in compressive strength tends to be constant when the steel member is strengthened with an increasing number of CFRP layers. For steel with a slenderness ratio of 120, this trend begins when 50 CFRP layers are used. Yet, the trend occurs earlier (smaller number of CFRP layers) as the slenderness ratio decreases (see Figure 12c). Comparing with the specimens model with a slenderness ratio of 100, for example, where the strength increase tends to be constant in the use of 40 layers of CFRP, the rate of increase in strength for the specimens model with a slenderness ratio of 70 has flattened in the use of only 20 CFRP layers. In line with the increasing strength, Figure 12b,d,f shows that such a non-linear trend also occurs in the rate of lateral displacement reduction. Based on these conditions, the authors believe that there is a certain number of CFRP layers (optimum layers) where the axial compression strength begins not to increase significantly, and the optimum layers will greatly depend on the dimensional parameters of the steel and the length of the CFRP.

### 4.2. Effect of Slenderness Ratio 

The slenderness ratio also highly affects the compressive strength increase of the members. The strength gain increases as the slenderness ratio is increased. This becomes very clear when a large number of CFRP layers are used. For example, for the use of 10-layer CFRP, the increase in compressive strength for members with slenderness ratios of 70 and 120 are only 6.25% and 11.4%, respectively (5.15% difference). However, when the layers are increased to 80, the strength gains of the members increase to be 13.7% and 68.4%, respectively (54.70% difference). However, as an increasing number of CFRP layers is used, a trend of the constant rate in strength increase can be observed, especially for members with a smaller slenderness ratio. Figure 12c summarizes the effect of slenderness ratios on the strength increase of members. 

### 4.3. Effect of CFRP Length 

The effects of CFRP length on the increase of ultimate load and lateral displacement reduction of the members can be seen in Figure 11a,c,e), which are then summarized in Figure 12a,b. It is clear from Figure 12a that the compressive strength increases along with the increasing CFRP length. This finding is in line with the experimental investigation results [21]. The difference of strength increases also becomes greater as the number of CFRP layers increased. The members with CFRP lengths of 385 mm and 625 mm are examples. Regarding the use of 10-layer CFRP, the strength increases of the members are 11.4% and 17.3% (5.90% difference), respectively. However, when the layers are increased to 80, the increase in strength changed to 68.4% and 112.7%, respectively (44.30% difference). The increase in lateral displacement reduction also occurs as the CFRP layers increased, but the difference between using smaller and greater number layers of CFRP is not large, as shown in Figure 12b (8.44% and 20.40% difference for using 10 and 80 CFRP layers, respectively). All of the above can be understood as results of decreasing the remaining length of the unstrengthened part of the steel while the CFRP are still within the required flexural rigidity for strengthening.

### 4.4. Effect of Different Values of CFRP’s Elastic Modulus 

The difference in the elastic modulus of CFRP is closely related to the fiber volume content (*V*_f_) and CFRP thickness. Theoretically, CFRP with a higher fiber volume content will have a lower thickness but higher elastic modulus. Conversely, CFRP with a smaller elastic modulus will have a higher thickness. The elastic modulus and thickness of CFRP will determine its flexural rigidity and contribution effect on strengthening the steel members. Figure 12e,f respectively demonstrate the effect of the elastic modulus of the CFRP (*E*1 = 62.63 GPa and *E*2 = 68.72 GPa) on the increase of compressive strength and lateral displacement reduction of steel members. It is confirmed that there is a very small increase in the strength gain and displacement reductions of steel members due to the decrease of CFRP’s elastic modulus from 68.72 to 62.63 GPa.

### 4.5. Strength Design for Strengthened Members 

**Figure 13:**
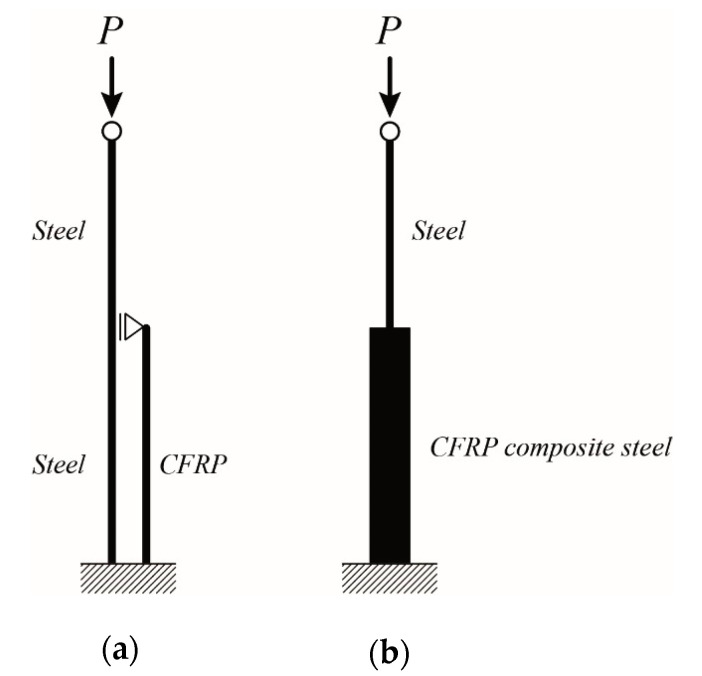
Structural model: (**a**) lower limit, (**b**) upper limit.

**Figure 14:**
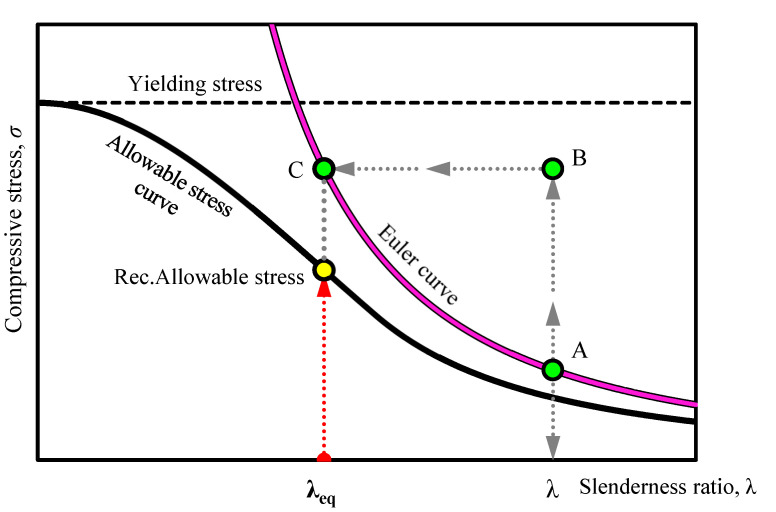
Determination of equivalent slenderness ratio (*λ*_eq_) and recommended allowable stress for strengthened members.

In this section, the method of equivalent slenderness ratio is used for determining the recommended allowable stress of the strengthened steel members. An outline of the steps for determining the slenderness ratio (*λ*_eq_), including the critical stress (Euler stress) for the lower and upper limit, has been clearly described in Yoresta et al. [21]. The lower and upper limits are respectively based on the assumption of fully unbonded and fully composite behavior between steel and the CFRP in a structure (See Figure 13). In summary, the procedure of the method follows the illustration shown in Figure 14. Bare steel with a slenderness ratio has a certain value of Euler stress (point A in Figure 14). The increased Euler stress of steel members due to unbonded CFRP strengthening (point B in Figure 14) will correspond to the elastic critical buckling stress (*F*_e_) in Equation (1) (Point C in Figure 14). By solving the equation, at this stage, the equivalent slenderness ratio has been obtained. Afterwards, the recommended allowable stress (*F*_cr_) is calculated according to the provisions used. In this study, the provision of the American Institute of Steel Construction (ANSI/AISC 360-16) is used; so, Equation (2) or Equation (3) will be involved, where the following are the properties of steel used in the current study: *F*_y_ is yield stress (328 MPa), *E* is elastic modulus (205 GPa), *L* is total length (560 mm, 800 mm, and 960 mm), *r* is radius of gyration (8 mm), and *k* = 1 (pinned-end members).

(1) $$F_{e}=\frac{\pi^{2}E}{{\left(kL/r\right)}^{2}}$$

(2) $$F_{cr}=\left(0.658^{F_{y}/F_{e}}\right)F_{y}\;\mathrm{for}\;\frac{kL}{r}\leq 4.71\sqrt{\frac{E}{F_{y}}}$$

(3) $$F_{cr}=0.877F_{e}\;\mathrm{for}\;\frac{kL}{r}>4.71\sqrt{\frac{E}{F_{y}}}$$

**Figure 15:**
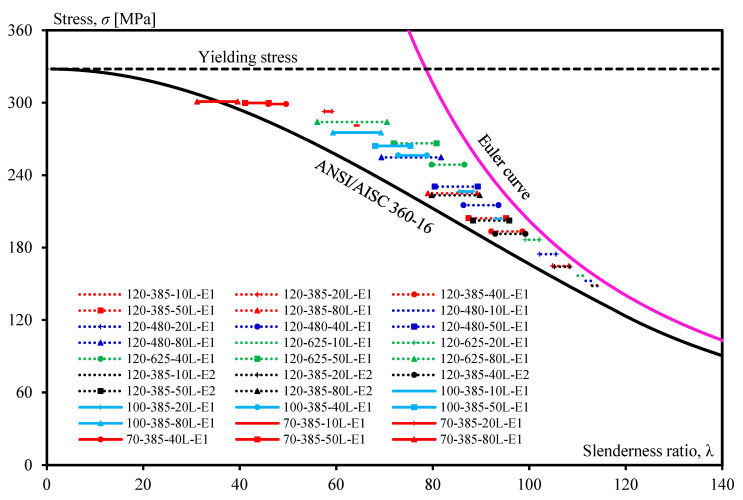
Compressive stress of strengthened steel members plotted based on an equivalent slenderness ratio.

The maximum stresses of all strengthened steel members obtained from finite element analysis are summarized in Table 4 and then plotted together with an allowable stress curve as well as Euler curve, as shown in Figure 15. The stresses are plotted within the range of the lower and upper limit of its recommended allowable compressive stress. It is clear from Figure 15 and Table 4 that the stresses lay between the allowable stress curve and Euler buckling curve with a partial safety factor calculated to be mostly less than 1. It is indicated that the method of the equivalent slenderness ratio can be applied to the design of axial compression steel members strengthened with unbonded CFRP laminates.

## 5. Conclusions

The unbonded CFRP method has been proposed for strengthening steel as an alternative to the adhesively bonded CFRP. This method relies on the flexural rigidity of CFRP, not the bonding strength between steel and CFRP. This method is much better, because no steel surface treatments are needed, so that the installation process becomes much faster and thus reduces the construction cost. In this paper, a numerical study on the use of unbonded CFRP for strengthening steel against buckling has been presented. A parametric study considering several parameters, i.e., the number of CFRP layers, CFRP length, slenderness ratio, and elastic modulus of CFRP, lead to producing a total of 33 FE results. Based on our investigation, the core findings can be summarized as follows:

1. The unbonded CFRP strengthening method is effective in improving the compressive strength of steel members as well as delaying overall buckling by reducing lateral displacements.
2. Increasing the number of CFRP layers will highly affect the increasing compressive strength of steel members.
3. The increase in compressive strength is higher for steel members with higher slenderness ratios.
4. A reduction in CFRP length will result in a decrease of the strength gain.
5. The difference in elastic modulus of CFRP only has a very small effect on strengthening steel with unbonded CFRP.
6. The method of the equivalent slenderness ratio proposed by the authors can be used for the design of axial compression steel members strengthened with unbonded CFRP laminates.

## Figures and Tables

**Table 1:** Specimen matrix.

Specimens	FE Model	CFRP Length (mm)	CFRP Thickness (mm)	Number of CFRP Layer	Elastic Modulus of CFRP (GPa)	Poisson’s Ratio of CFRP
NS56BL	NS56BL-FE	-	-	-	-	-
BL56F26-1	BL56F26-1-FE	260	5.30	26	68.72	0.0311
BL56F26-2	BL56F26-2-FE	260	5.85	28	67.49	0.0312
BL56F34-1	BL56F34-1-FE	340	5.43	27	69.94	0.0311
BL56F34-2	BL56F34-2-FE	340	6.02	28	65.06	0.0313

**Table 2:** Comparison between FE analysis and test results.

Specimens	FE Model	Ultimate Load (kN)		*P*_exp._/*P*_FE_
		Exp. (*P*_exp._)	FE (*P*_FE_)	
NS56BL	NS56BL-FE	214	213	1.00
BL56F26-1	BL56F26-1-FE	249	230	1.08
BL56F26-2	BL56F26-2-FE	228	231	0.99
BL56F34-1	BL56F34-1-FE	250	235	1.06
BL56F34-2	BL56F34-2-FE	234	236	0.99

**Table 3:** Parametric study results.

FE Model	Maximum Load (kN)	Increase Over Unstrengthened Model (%)	Lateral Displacement at Maximum Load (mm)	Decrease Over Unstrengthened Model (%)
Control-120	107.4	-	6.57	-
120-385-10L-E1	119.7	11.4	6.03	8.16
120-385-20L-E1	132.6	23.4	5.69	13.3
120-385-40L-E1	155.5	44.8	3.73	43.2
120-385-50L-E1	164.3	53.0	2.91	55.7
120-385-80L-E1	181.0	68.4	2.05	68.7
120-480-10L-E1	122.6	14.1	5.53	15.8
120-480-20L-E1	140.4	30.7	5.10	22.3
120-480-40L-E1	173.1	61.1	3.35	48.9
120-480-50L-E1	185.4	72.6	2.47	62.3
120-480-80L-E1	204.9	90.7	1.35	79.5
120-625-10L-E1	126.1	17.3	5.48	16.6
120-625-20L-E1	150.0	39.6	4.72	28.1
120-625-40L-E1	200.0	86.2	2.60	60.4
120-625-50L-E1	214.2	99.4	1.70	74.1
120-625-80L-E1	228.5	112.7	0.71	89.1
120-385-10L-E2	119.3	11.0	6.39	2.69
120-385-20L-E2	132.0	22.9	5.19	21.0
120-385-40L-E2	153.9	43.3	4.04	38.5
120-385-50L-E2	162.8	51.6	3.15	52.0
120-385-80L-E2	179.7	67.2	2.16	67.1
Control-100	146.6	-	3.44	-
100-385-10L-E1	164.0	11.9	3.31	3.76
100-385-20L-E1	182.1	24.2	2.68	22.0
100-385-40L-E1	206.2	40.7	1.52	55.8
100-385-50L-E1	212.5	45.0	1.13	67.2
100-385-80L-E1	221.4	51.0	0.82	76.2
Control-70	212.8	-	0.87	-
70-385-10L-E1	226.1	6.25	0.73	16.0
70-385-20L-E1	235.5	10.6	0.60	30.5
70-385-40L-E1	240.4	12.9	0.39	55.0
70-385-50L-E1	241.1	13.3	0.35	59.9
70-385-80L-E1	242.0	13.7	0.25	71.3

**Table 4:** Strength recommendation for strengthened members.

FE Model	Max. Stress (MPa)	Euler Buckling Stress (MPa)		*λ*eq. (Low–Up)	Rec. Design Strength (MPa) (Low–Up)	*σ*(rec.)/*σ*(FEM)	
		Low	Up			Low	Up
120-385-10L-E1	148.8	155.7	160.1	114.0–112.4	135.81–139.12	0.91	0.94
120-385-20L-E1	164.8	173.1	184.1	108.1–104.8	148.39–155.59	0.90	0.94
120-385-40L-E1	193.4	208.2	238.5	98.58–92.10	169.63–184.46	0.88	0.95
120-385-50L-E1	204.3	223.3	264.7	95.19–87.43	177.36–195.26	0.87	0.96
120-385-80L-E1	225.0	254.4	324.2	89.18–79.00	191.22–214.76	0.85	0.95
120-480-10L-E1	152.4	159.2	163.8	112.7–111.1	138.45–141.88	0.91	0.93
120-480-20L-E1	174.6	181.7	194.1	105.5–102.1	154.05–161.72	0.88	0.93
120-480-40L-E1	215.2	230.9	271.2	93.62–86.37	180.97–197.71	0.84	0.92
120-480-50L-E1	230.5	253.5	312.9	89.33–80.41	190.86–211.52	0.83	0.92
120-480-80L-E1	254.8	302.9	420.5	81.73–69.36	208.47–236.64	0.82	0.93
120-625-10L-E1	156.7	164.0	168.0	111.1–109.8	142.01–144.84	0.91	0.92
120-625-20L-E1	186.5	194.3	205.9	102.1–99.13	161.80–168.39	0.87	0.90
120-625-40L-E1	248.7	270.0	318.0	86.56–79.77	197.28–212.99	0.79	0.86
120-625-50L-E1	266.4	309.9	391.3	80.80–71.91	210.62–230.94	0.79	0.87
120-625-80L-E1	284.1	406.9	643.4	70.52–56.08	234.07–264.98	0.82	0.93
120-385-10L-E2	148.3	155.4	159.7	114.1–112.6	135.58–138.84	0.91	0.94
120-385-20L-E2	164.1	172.1	182.7	108.4–105.2	147.71–154.69	0.90	0.94
120-385-40L-E2	191.4	205.6	234.2	99.20–92.95	168.22–182.51	0.88	0.95
120-385-50L-E2	202.5	220.2	259.2	95.85–88.35	175–193.12	0.87	0.95
120-385-80L-E2	223.4	251.3	317.9	89.73–79.78	189.95–212.97	0.85	0.95
100-385-10L-E1	203.9	228.3	234.9	94.14–92.80	179.76–182.86	0.88	0.90
100-385-20L-E1	226.5	259.3	277.0	88.34–85.47	193.15–199.80	0.85	0.88
100-385-40L-E1	256.4	326.0	381.5	78.79–72.83	215.26–228.87	0.84	0.89
100-385-50L-E1	264.3	356.3	436.8	75.36–68.06	223.12–239.53	0.84	0.91
100-385-80L-E1	275.3	421.6	575.4	69.28–59.30	236.83–258.38	0.86	0.94
70-385-10L-E1	281.2	485.0	495.7	64.59–63.89	247.14–248.65	0.88	0.88
70-385-20L-E1	292.8	579.2	611.0	59.10–57.54	258.79–262.00	0.88	0.89
70-385-40L-E1	298.9	823.0	960.8	49.58–45.89	277.61–284.33	0.93	0.95
70-385-50L-E1	299.8	956.2	1198.2	46.00–41.09	284.13–292.49	0.95	0.98
70-385-80L-E1	301.0	1291.4	2083.4	39.58–31.16	294.92–307.08	0.98	1.02
